# Effect of N-Feruloylserotonin and Methotrexate on Severity of Experimental Arthritis and on Messenger RNA Expression of Key Proinflammatory Markers in Liver

**DOI:** 10.1155/2016/7509653

**Published:** 2016-07-31

**Authors:** Ľudmila Pašková, Viera Kuncírová, Silvester Poništ, Danica Mihálová, Radomír Nosáľ, Juraj Harmatha, Iveta Hrádková, Tomáš Čavojský, František Bilka, Katarína Šišková, Ingrid Paulíková, Lýdia Bezáková, Katarína Bauerová

**Affiliations:** ^1^Department of Cell and Molecular Biology of Drugs, Faculty of Pharmacy, Comenius University, 832 32 Bratislava, Slovakia; ^2^Institute of Experimental Pharmacology and Toxicology, Slovak Academy of Sciences, 841 04 Bratislava, Slovakia; ^3^Institute of Organic Chemistry and Biochemistry, Academy of Sciences of the Czech Republic, 166 10 Prague, Czech Republic; ^4^Faculty of Food and Biochemical Technology, University of Chemistry and Technology, 166 28 Prague, Czech Republic; ^5^Department of Galenic Pharmacy, Faculty of Pharmacy, Comenius University, 832 32 Bratislava, Slovakia

## Abstract

Rheumatoid arthritis (RA) is a chronic inflammatory disease, leading to progressive destruction of joints and extra-articular tissues, including organs such as liver and spleen. The purpose of this study was to compare the effects of a potential immunomodulator, natural polyphenol N-feruloylserotonin (N-f-5HT), with methotrexate (MTX), the standard in RA therapy, in the chronic phase of adjuvant-induced arthritis (AA) in male Lewis rats. The experiment included healthy controls (CO), arthritic animals (AA), AA given N-f-5HT (AA-N-f-5HT), and AA given MTX (AA-MTX). N-f-5HT did not affect the body weight change and clinical parameters until the 14th experimental day. Its positive effect was rising during the 28-day experiment, indicating a delayed onset of N-f-5HT action. Administration of either N-f-5HT or MTX caused reduction of inflammation measured as the level of CRP in plasma and the activity of LOX in the liver. mRNA transcription of TNF-*α* and iNOS in the liver was significantly attenuated in both MTX and N-f-5HT treated groups of arthritic rats. Interestingly, in contrast to MTX, N-f-5HT significantly lowered the level of IL-1*β* in plasma and IL-1*β* mRNA expression in the liver and spleen of arthritic rats. This speaks for future investigations of N-f-5HT as an agent in the treatment of RA in combination therapy with MTX.

## 1. Introduction

Rheumatoid arthritis (RA) is a chronic systemic inflammatory disease affecting predominantly joints, synovial membranes, articular cartilages, and subchondral bones [1]. Disease progression is attributed to increases in reactive oxygen species (ROS) and oxidative stress (OS) in the lesion sites [2]. Proinflammatory cytokines, such as tumor necrosis factor-*α* (TNF-*α*), interleukin-1*β* (IL-1*β*), and IL-6, regulate the inflammatory and immune responses and play a pivotal role in the disease [3]. Overproduction of nitric oxide (NO), as a result of induction of inducible nitric oxide synthase (iNOS) due to enhanced production of these cytokines, is associated with persistent inflammation and tissue destruction in experimental arthritis models, including rheumatoid arthritis [4, 5]. A number of inflammation stimuli, including TNF-*α*, IL-1*β*, IL-6, or ROS, can activate proinflammatory pathways involved in RA pathogenesis, concerning predominantly nuclear factor-*κ*B (NF-*κ*B), mitogen activated protein kinases (MAPKs), or Janus kinases/signal transducers and activators of transcription (JAK/STAT1/3) [6–8]. This results in translocation of relevant downstream transcription factors from the cytoplasm to the nucleus, where they activate messenger RNA (mRNA) expression of target genes, including IL-1*β*, TNF-*α*, iNOS, and 12/15-lipoxygenase (LOX), leading to overproduction of corresponding proteins. Cytokines released into the synovium reach also the systemic circulation and act in other tissues and organs such as lungs, vascular tissue, liver, and heart [9]. Several recent investigations reported damage of vital organs with various degrees of impairment, considered to be secondary complications of RA and a major predictor of mortality in RA patients [10]. Increasing evidence is pointing to the critical role of the liver in modulating the immune response in autoimmune and chronic inflammatory diseases including RA [5, 11, 12]. The hepatic biochemical and immunological alterations are associated with and influenced by changes in the oxidative state of liver cells [5].

Adjuvant-induced arthritis (AA) in rats not only is an experimental model of polyarthritis but also induces pathological changes in a variety of other tissues, including the liver and spleen [13]. It is a useful tool to study immunopathologic processes, autoimmune chronic inflammation, and inflammatory cachexia in rodents. In addition, at the molecular level, mRNA profiling suggests that this model is also similar to human RA, particularly in tissue gene expression and in the activation of regulatory pathways [11, 14].

Numerous studies reported natural polyphenols as potential therapeutic agents of diseases caused by OS and inflammation [15–17]. N-feruloylserotonin (N-f-5HT, N-feruloyl-5-hydroxy-tryptamine) is a conjugated serotonin, a member of the indole hydroxycinnamic acid amides, with serotonin (5-HT) and ferulic acid (fa) as representative components of its structure. Hydroxycinnamic acid amides of serotonin, synthesized by serotonin N-hydroxycinnamoyltransferase, are present in several vegetables [18] and wild-growing plants whose seeds are used in herbal medicine in Eastern countries [18–20]. In cell-based studies, under short-term high-glucose conditions, N-f-5HT exerted an inhibitory effect on overproduction of mitochondrial superoxide by acting as scavenger of superoxide [21]. N-f-5HT attenuated the upregulation of mRNA and proteins of ROS-dependent adhesion (vascular cell adhesion protein-1 (VCAM-1)) and migration factors (monocyte chemoattractant protein-1 (MCP-1)), crucial in early atherosclerosis lesions in human aortic endothelial cells, and inhibited the activation of transcription factor NF-*κ*B [21]. Furthermore, N-f-5HT showed a protective effect on ROS-related neuronal damage by decreasing the activity of proapoptotic caspase-3 [22]. N-f-5HT isomers isolated from seeds of* Leuzea carthamoides* were shown to inhibit protein kinase C *α*/*β* II activation and decrease the oxidative burst of human whole blood and isolated neutrophils* in vitro* [23]. N-f-5HT was also found to have a protective effect against LDL oxidation and atherogenesis in experimental animals and in human studies [24–26].

Methotrexate (MTX), used as a standard drug in our study, represents the most frequently used pharmacotherapy of RA in clinical practice. Its administration is, however, limited due to its toxic side effects [27, 28]. Yet application of a combination therapy of MTX with other potential immunomodulators, synthetic drugs [29] or natural substances [30–32], might elevate the therapeutic efficacy: decrease the dose of MTX and thus its side effects. In our previous study, we showed that administration of N-f-5HT to MTX-treated arthritic rats lowered the dose of MTX for the required sustained antirheumatic impact [33]. In this study, we focused on the therapeutic impact of N-f-5HT and MTX administered in monotherapy and on details of the inflammatory state in the arthritic rat liver with the aim to elucidate the molecular mechanisms of their effect. One of the possible clarifying approaches is to study the mRNA expression of key proinflammatory markers (IL-1*β*, TNF-*α*, and iNOS) in the liver of treated and untreated arthritic rats. Further, it is of particular interest to expand our knowledge on the effect of N-f-5HT and MTX in the AA model, which in turn should allow extrapolations of these results to RA patients. To this aim we evaluated also conventional arthritic parameters (HPV, arthritic score, body weight change, and weight of the liver) along with changes in plasmatic levels of IL-1*β* and CRP and the activity of 12/15-LOX in the liver.

## 2. Materials and Methods

### 2.1. Animals

Adult male Lewis rats weighing 160–180 g were obtained from Charles River Wiga, Germany. The rats had free access to standard pelleted diet and tap water. The experimental protocol was approved by the Ethics Committee of the Institute of Experimental Pharmacology and Toxicology and by the Slovak State Veterinary and Food Administration in accordance with the European Convention for the Protection of Vertebrate Animals Used for Experimental and Other Scientific Purposes and was in line with Slovak legislation.

### 2.2. Induction of Adjuvant Arthritis

To induce a rat model of adjuvant arthritis (AA), rats were intradermally injected with a suspension of heat-inactivated* Mycobacterium butyricum* in incomplete Freund's adjuvant (Difco Laboratories, Detroit, MI, USA). The injection was performed near the tail base on the first experimental day.

### 2.3. Experimental Design and Animal Treatments

The experiments included 4 groups of animals.

*Group 1*. The first group comprised healthy control rats (CO).

*Group 2*. The second group comprised untreated adjuvant arthritis rats (AA).

*Group 3*. The third group comprised adjuvant arthritis rats treated with methotrexate (Methotrexat® EBEWE* sol inj* 20 mg/2.0 mL) in oral dose of 0.4 mg/kg twice a week (AA-MTX).

*Group 4*. The fourth group comprised adjuvant arthritis rats treated with N-feruloylserotonin dissolved in suspension of methylcellulose Tween 80 at a dose of 3 mg/kg/day orally (AA-N-f-5HT).

Drugs were administered orally by gastric gavage from day 0 (the day of treatment) to day 28 of the study. Blood for plasma preparation was taken by retroorbital puncture on day 14 and by cardiac puncture on day 28 under deep ketamine/xylazine anesthesia. After the animals had been sacrificed under deep ketamine/xylazine anesthesia, tissues for liver and spleen homogenate preparation were taken at the end of the experiment (day 28). Blood in heparinized tubes for plasma preparation was centrifuged at 3000 rpm for 15 minutes at 4°C. Samples were stored at −80°C until biochemical and immunological analysis.

Fraction of four isomers of N-f-5HT (Table 1) was isolated from the seeds of* Leuzea carthamoides* (Wild) DC by solvent extraction. This was then followed by column chromatography on silica gel and HPLC separations under conditions previously reported [35, 36].

### 2.4. Change of Hind Paw Volume (HPV)

The hind paw volume (HPV) was recorded on days 14, 21, and 28 with the use of an electronic water plethysmometer (UGO BASILE, Comerio, Varese, Italy). Calculation of the increase in hind paw volume in mL assessed the intensity of the edema.

### 2.5. Arthritic Score

The arthritic score was measured as the total score of HPV (mL, max. points 8) + paw diameter of forelimb (mm, max. points 5) + diameter of scab in the site of MB application, measured in parallel to the spinal column (mm, max. points 5) for each animal on all experimental days monitored [33].

### 2.6. Body Weight Change

Body weight change (BWC; g) was measured on days 1, 14, 21, and 28. BWC was calculated as the difference of the body mass measured on days 14, 21, and 28 to the body weight measured at the beginning of the experiment (day 1).

### 2.7. Measurement of C-Reactive Protein (CRP) in Plasma

For the determination of rat CRP concentration in plasma (*μ*g/mL), the ELISA kit from Immunology Consultant Laboratories, Inc. was used. The reaction of secondary biotin-conjugated anti-rat CRP antibody was evaluated by streptavidin-HRP. The tetramethylbenzidine reaction with HRP bound to immune complex was measured at 450 nm (microplate reader, Labsystems Multiskan RC). The results were calculated using the standard calibration curve on internal standards.

### 2.8. Measurement of Interleukin-1*β* (IL-1*β*) in Plasma

For the determination of IL-1*β* concentration in plasma, the ELISA kit from R&D Systems Quantikine® was used. The assay procedures followed the description in the product manual. Rat cytokine present in the samples binds to anti-rat cytokine antibodies absorbed in the microwells. The reaction of secondary biotin-conjugated anti-rat cytokine antibody is evaluated by HRP. The tetramethylbenzidine reaction with HRP bound to immune complex was measured at 490 nm in comparison with the reference wavelength of 620 nm (microplate reader MRX II). The results were calculated using the standard calibration curve on internal standards.

### 2.9. Tissue Activity of 12/15-Lipoxygenase (LOX) in Liver

Concentration of proteins in liver homogenates was determined by using the Bradford method [37] and expressed in mg/mL of enzyme preparation (cytosolic fraction from rat lung and liver tissues). Linoleic acid (99%, Sigma-Aldrich, USA) was used as a substrate prepared in solubilized state as described [38] in the concentration of 0.2143 × 10^−5^–0.7143 × 10^−5^ M. The assay of LOX was monitored for 60 seconds as an increase in the absorbance at 234 nm, reflecting the formation of hydroperoxylinoleic acid. For the LOX activity assay, an UV/VIS Spectrometer Perkin-Elmer Lambda 35 (USA) was used. The reaction medium contained a 50 mM Tris-HCl buffer (pH 7.0), 2.5 *μ*L of the enzyme, and solubilized linoleic acid.

### 2.10. Total RNA Isolation and Quantitative RT-PCR

Total RNA was isolated from the rat liver and spleen using RNAzol RT (Sigma-Aldrich) and converted into complementary DNA (cDNA) using the PrimeScript RT Reagent Kit (Takara) following the protocols of the manufacturers. Amplification and detection of cDNA of reference and target genes were performed on a 7300 Real-Time PCR System (Applied Biosystems) using HOT FIREPol EvaGreen® qPCR Mix Plus (ROX) (Solis BioDyne). Relative mRNA expressions of IL-1*β*, TNF-*α*, and iNOS were analyzed using the ΔΔCt value method [39]. PCR products were evaluated by melting curve analysis to confirm the specific amplification. *β*-actin was used as a reference gene. The sequences of the primers were designed and checked using Primer3 and Oligo Analyzer 1.0.3 (Table 2).

### 2.11. Statistical Analyses

Mean and SEM values were calculated for each parameter in each group (8–10 animals in each experimental group). All measurements were done in duplicate or triplicate. Statistically significant differences among treated, untreated, and control groups were tested using parametric Analysis of Variance (ANOVA).* Post hoc* tests (Tukey-Kramer (ANOVA)) were applied in situations where differences among groups were significant at the level of significance *α* = 0.05. After* post hoc* testing, the following significance levels were specified: extremely significant (^*∗∗∗*^ *p* < 0.001), highly significant (^*∗∗*^ *p* < 0.01), significant (^*∗*^ *p* < 0.05), and not significant (*p* > 0.05).

## 3. Results and Discussion

### 3.1. Effect of N-f-5HT on Clinical Parameters: Arthritic Score and Change of Hind Paw Volume (HPV) and Parameters of Cachexia

Antioxidant properties of polyphenols including N-f-5HT have been reported [21, 40, 41]. Nevertheless, the N-f-5HT impact on chronic inflammatory and OS-inducing arthritis, which could widen the possibilities of the RA therapy, remains to be elucidated. In our previous study in the model of AA, N-f-5HT in the dosage of 15 mg/kg markedly potentiated the therapeutic effect of low-dose (nontherapeutic dose) MTX (0.3 mg/kg) on arthritic (hind paw volume and arthritic score) and inflammatory parameters (IL-17, MCP-1, and CRP), yet it resulted in insignificant effect in monotherapy [33]. As data about the optimal N-f-5HT dose in the rat model are scarce, we decided to study two doses of N-f-5HT: (i) when 15 mg/kg exceeded the physiologically acceptable concentration, we used 3 mg/kg, and (ii) when 15 mg/kg was too low to reach the maximal effect, we used 30 mg/kg. Unexpectedly, contrary to the lower dose of N-f-5HT, the higher dose exhibited minor effect on the parameters examined and/or these varied strongly among the animals. For this reason, this report shows only the data evaluating the lower dose of N-f-5HT. In this study, we used the therapeutic dose of MTX (0.4 mg/kg) with the intention to compare each mechanism of action of MTX and N-f-5HT, both evaluated in monotherapy.

The significant rise in arthritic parameters, arthritic score, and HPV confirmed the arthritis in our model in rats. The arthritic score showed an increase in the untreated arthritic group compared to the control group on all days monitored (AA versus CO, day 14, ^*∗∗*^ *p* < 0.01; day 21 and day 28, ^*∗∗∗*^ *p* < 0.001; Table 3). At the end of the experiment, the arthritic score was almost doubled in the AA group compared to controls. A trend toward reduction was observed after administration of N-f-5HT to AA animals on day 28, but the effect was not statistically significant. The treatment with MTX significantly reduced the arthritic score on observation days 21 and 28, compared to the untreated arthritic group, proving the therapeutic potential of the applied dose of MTX (AA-MTX versus AA, day 21, ^+^ *p* < 0.05; day 28, ^++^ *p* < 0.01; Table 3).

Similarly, the change in HPV showed an increase in the untreated arthritic group compared to the control group on days 21 and 28 (AA versus CO, day 21, ^*∗∗*^ *p* < 0.01; day 28, ^*∗*^ *p* < 0.05; Table 3). The administration of N-f-5HT induced no modification of HPV of the arthritic animals on any day monitored. MTX therapy significantly reduced the observed swelling on days 21 and 28 compared to the untreated arthritic group (AA-MTX versus AA, day 21 and day 28, ^+++^ *p* < 0.001; Table 3).

The muscle wasting condition due to high catabolic activity, known as rheumatoid cachexia, occurring in approximately two-thirds of all patients with RA, is mediated by TNF-*α* and IL-1*β* in RA [42]. Papers published over the past years confirmed that oxidative metabolism was considerably enhanced in the liver of adjuvant-induced arthritis in rats [43–46]. Rats used in this study revealed signs of cachexia (Table 3). A significant decrease in body weight change (BWC) was observed on all experimental days in the AA group. The BWC of the arthritic rats was 56% on day 14, 19% on day 21, and 27% on day 28 (AA versus CO, days 14, 21, and 28, ^*∗∗∗*^ *p* < 0.001; Table 3) of the BWC of healthy controls. N-f-5HT treatment led to a significant increase of BWC on day 28 (AA-N-f-5HT versus AA, ^+^ *p* < 0.05; Table 3). Administration of MTX elevated the BWC, yet the effect was not significant.

No difference was noted in the fresh weight of the liver. These results are comparable with reported manifestations in this experimental arthritis model [5]. The administration of N-f-5HT in arthritic animals did not change these parameters on any of the days observed. The liver weights were significantly lower (AA-MTX versus AA, ^+^ *p* < 0.05; Table 3) only in the group of rats treated with MTX. The reduced weight of the liver in MTX-treated rats was assumed to be the result of inhibition of the pathway of* de novo* DNA synthesis by MTX [47, 48].

In summary, the statistical significance of 3 mg/kg of N-f-5HT treatment was determined only for BWC. The arthritic score revealed a trend toward the positive effect increasing with time, indicating a late onset of N-f-5HT action (Table 3). As expected, significant differences were found in the arthritic score and HPV in the arthritic animals treated with the therapeutic dose of 0.4 mg/kg MTX compared to those treated with the low dose of 0.3 mg/kg MTX [33].

### 3.2. Effect of N-f-5HT and MTX on IL-1*β* Plasmatic Level Measured on Day 14

**Figure 1:**
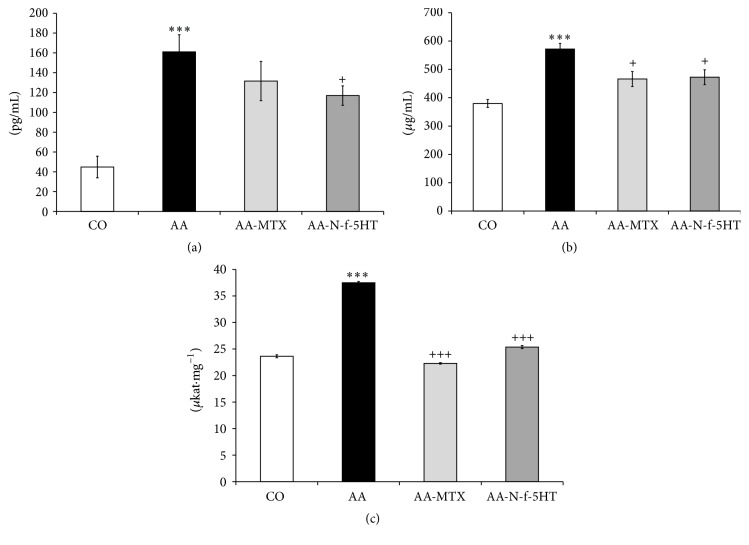
(a) Level of IL-1*β* in plasma in pg/mL measured on day 14. (b) Level of CRP in plasma in *μ*g/mL measured on day 28. (c) 12/15-LOX activity in *μ*kat·mg^−1^ in liver. CO, control group; AA, adjuvant arthritis group; AA-N-f-5HT, adjuvant arthritis group given N-feruloylserotonin; AA-MTX, adjuvant arthritis group given methotrexate. Results are mean ± SEM; *n* = 8–10. The symbols *∗* and + show significant difference: ^*∗∗∗*^ *p* < 0.001 versus CO, ^+^ *p* < 0.05 versus AA, and ^+++^ *p* < 0.001 versus AA.

IL-1*β*, a prototypic proinflammatory cytokine, is a major mediator of the inflammatory cascade in RA, which is involved in the mechanisms leading to progressive joint destruction [3]. In the model of AA, the early phases of the disease seem to be characterized by a systemic increase of IL-1*β* [49]. The inflammatory process in AA is then self-remitting with time [50]. The plasmatic level of IL-1*β*, a protein of multiorgan origin, was significantly increased in arthritic animals compared to the control group in the early phase of AA, on day 14 (AA versus CO, ^*∗∗∗*^ *p* < 0.001; Figure 1(a)), ascertaining the presence of inflammation. Administration of MTX did not lead to a significant change of plasmatic IL-1*β* concentration; only a trend toward reduction was observed on day 14. It is noteworthy that N-f-5HT treatment resulted in a significant decrease of IL-1*β* level in plasma (AA-N-f-5HT versus AA, ^+^ *p* < 0.05; Figure 1(a)). This result is interesting, as this molecule was reported to be relevant in driving the transition from the acute phase to the chronic irreversible phase of the disease and it has been suggested that it could be the target of early intervention to stop the course toward the chronic form of the disease [49]. The blocking IL-1*β* protects bone and cartilage from progressive destruction in RA and its inhibition could be effective in the treatment of this disease [7].

### 3.3. Effect of N-f-5HT and MTX on C-Reactive Protein (CRP) Level in Plasma on Day 28

The AA model represents a model of polyarthritis, which expands to systemic inflammation [13]. Activation of T and B cells, macrophages, and inflammatory mediators TNF-*α*, IL-1*β*, and IL-6 aggravates the oxidative damage of the vital organs in rheumatoid arthritis, such as the liver. The liver, in turn, influences the systemic inflammation via producing inflammatory cytokines and mediators such as TNF-*α*, IL-1*β*, IL-6, NO, CRP, and LOX. IL-6, IL-1*β*, and TNF-*α* promote the synthesis of CRP in hepatocytes via STAT3 [51, 52] and NF-*κ*B [53] pathways. The level of the systemic inflammatory parameter CRP in plasma, resulting from liver synthesis, was increased significantly in the group of arthritic animals compared with control animals in the chronic phase of the disease on experimental day 28 (AA versus CO, ^*∗∗∗*^ *p* < 0.001; Figure 1(b)). Administration of N-f-5HT and MTX significantly reduced the plasmatic levels of CRP on day 28 compared to the untreated group of arthritic animals (AA-N-f-5HT versus AA, ^+^ *p* < 0.05; AA-MTX versus AA, ^+^ *p* < 0.05; Figure 1(b)). Interaction of CRP with Fc-gamma receptors (Fc*γ*R) Fc*γ*RI and Fc*γ*RIIA is known to promote the production of proinflammatory cytokines, resulting in the amplification loop of inflammatory reaction [54]. These processes are initiated through the induction of the receptor activator of nuclear factor-*κ*B ligand (RANKL) protein and direct stimulation of osteoclastogenesis, causing a loop between inflammation and bone destruction in RA. CRP enhances both the proinflammatory response and bone destruction. In the treatment of RA, a lowered CRP level thus not only is a significant parameter in terms of disease progression elimination but also has a direct impact on decreasing the degree of bone destruction [55].

### 3.4. Effect of N-f-5HT and MTX on 12/15-LOX Activity in the Liver

Alterations in the oxidative state lead to the activation of NF-*κ*B and NF-*κ*B-dependent genes, such as LOX. The enzyme 5-LOX catalyzes the conversion of arachidonic acid to leukotrienes, whose production has been associated with inflammation in arthritis. Suppression of 5-LOX expression ameliorates clinical parameters in RA and AA [56, 57]. A similar role can be attributed to 15-LOX [58]. Increased levels of NF-*κ*B in the lung and liver as well as increased activity of LOX in the lung highlight the importance of extra-articular manifestations of AA [38]. In our experiment, liver 12/15 LOX activity increased in arthritic animals in comparison to healthy animals (AA versus CO, ^*∗∗∗*^ *p* < 0.001; Figure 1(c)). The effect of N-f-5HT on the activity of 12/15-LOX in liver homogenate was comparable with that of MTX. After administration of MTX or N-f-5HT, a significant decrease to control levels was assessed in the liver of the AA group (AA-N-f-5HT versus AA, ^+++^ *p* < 0.001; AA-MTX versus AA, ^+++^ *p* < 0.001; Figure 1(c)). Thus the anti-inflammatory effect of N-f-5HT in AA was supported by the ability of the molecule to inhibit 12/15-LOX activity. Similar to this result, recent observations also reported that several other flavonoids may act as LOX inhibitors [59].

### 3.5. Effect of N-f-5HT and MTX on mRNA Expression of iNOS and TNF-*α* in the Liver

**Figure 2:**
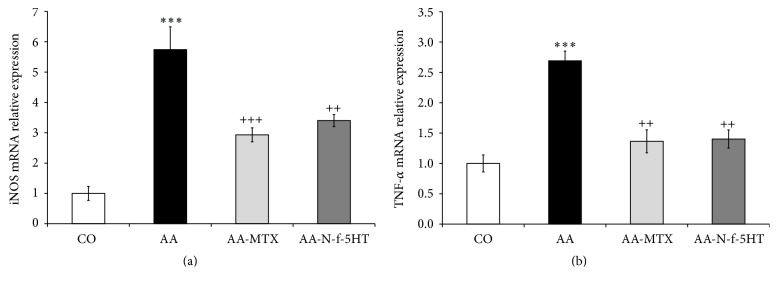
Relative changes of iNOS (a) and TNF-*α* (b) mRNA expressions normalized to *β*-actin mRNA in the rat liver. Control was preset at 1. CO, control group; AA, adjuvant arthritis group; AA-N-f-5HT, adjuvant arthritis group given N-feruloylserotonin; AA-MTX, adjuvant arthritis group given methotrexate. The results are given as average ± SEM; *n* = 8–10. The symbols *∗* and + show significant difference: ^*∗∗∗*^ *p* < 0.001 versus CO; ^++^ *p* < 0.01 versus AA; ^+++^ *p* < 0.001 versus AA.

In AA, the gene expression levels of TNF-*α* and iNOS produced in the liver were reported to increase [60, 61]. Also, in our study, the levels of TNF-*α* and iNOS mRNA expressions were significantly increased in arthritic animals (both ^*∗∗∗*^ *p* < 0.001, AA versus CO; Figures 2(a) and 2(b)). It was proposed that these modifications in the liver of arthritic rats not only were a consequence of the metabolic alterations caused by the disease, especially the increased oxidative metabolism [17], but also depended on increased inflammatory parameters in the liver. The same agents that increase oxidative metabolism, TNF-*α*, IL-1*β*, IL-6, and others [62], are responsible for increasing the activity of iNOS in several tissues. An increase of iNOS activity as a consequence of elevated iNOS mRNA expression was considered to play a dominant role in the pathogenesis of RA [4]. NO generation by iNOS induced in chondrocytes in the initial stage of AA may play a key role in triggering the subsequent events in arthritis [4]. In general, the use of NOS inhibitors has been shown to exert beneficial effects in experimentally induced arthritis [63]. However, which types of cells expressing iNOS are associated with the induction or progression of adjuvant-induced arthritis via NO generation remains uncertain. mRNA expression of iNOS in rat liver was reduced following MTX (AA-MTX versus AA, ^+++^ *p* < 0.001; Figure 2(a)) and N-f-5HT treatment (AA-N-f-5HT versus AA, ^++^ *p* < 0.01; Figure 2(a)).

The effect of MTX treatment on TNF-*α* protein and mRNA expression differs among studies, depending on the conditions of the given study, concerning gender of patients, type of cell line, duration of treatment, MTX dose, and so forth [64]. In our study in the rat AA model, administration of MTX attenuated significantly the mRNA expression of TNF-*α* (AA-MTX versus AA, ^++^ *p* < 0.01; Figure 2(b)). In many patients, however, MTX treatment does not result in lower TNF-*α* plasma concentration. When MTX fails to produce an adequate response, newer therapies are used in combination with MTX. Blocking TNF-*α* with anti-TNF-*α* monoclonal antibodies significantly decreased the signs and symptoms of RA compared to placebo in RA patients with active disease receiving MTX [65, 66]. Thus, the N-f-5HT-driven significant reduction of TNF-*α* mRNA expression (A-N-f-5HT versus AA, ^++^ *p* < 0.01; Figure 2(b)) suggests an intriguing effect on RA treatment, calling for deeper investigation.

### 3.6. Effect of N-f-5HT and MTX on mRNA Expression of IL-1*β* in Liver and Spleen

**Figure 3:**
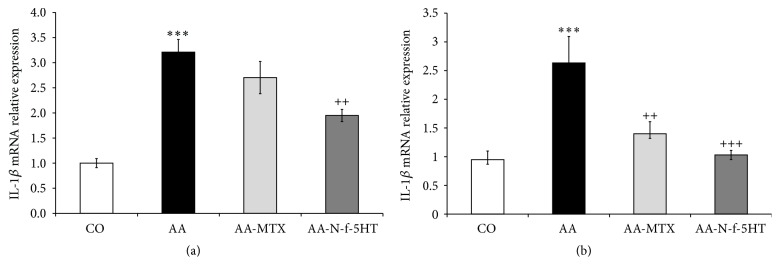
Relative changes of IL-1*β* mRNA expression normalized to *β*-actin mRNA in the liver (a) and in the spleen (b). Control was preset at 1. CO, control group; AA, adjuvant arthritis group; AA-N-f-5HT, adjuvant arthritis group given N-feruloylserotonin; AA-MTX, adjuvant arthritis group given methotrexate. The results are given as average ± SEM; *n* = 8–10. The symbols *∗* and + show significant difference: ^*∗∗∗*^ *p* < 0.001 versus CO; ^++^ *p* < 0.01 versus AA; ^+++^ *p* < 0.001 versus AA.

Increase of mRNA expression was observed for IL-1*β* in the liver of arthritic animals (AA versus CO, ^*∗∗∗*^ *p* < 0.001; Figure 3(a)) as expected [60]. Administration of MTX did not lead to significant attenuation of IL-1*β* transcription in the liver. This is in concert with previous studies of MTX function in different types of cells (e.g., human peripheral blood mononuclear cells and murine peritoneal and splenic cells) [67, 68]. On the other hand, MTX exhibits another mechanism of IL-1*β* function inhibition, which involves blocking the binding of IL-1*β* to IL-1*β* receptor in the membrane of peripheral blood cells (monocytes, lymphocytes, and granulocytes) [69]. Contrary to MTX, treatment with N-f-5HT led to a substantial inhibition of IL-1*β* gene expression (AA-N-f-5HT versus AA, ^++^ *p* < 0.01; Figure 3(a)).

Further, we examined IL-1*β* mRNA expression in the main immunocompetent organ, in the rat arthritic spleen, which has not been studied previously in terms of the AA model, related to IL-1*β* expression. We observed IL-1*β* mRNA expression activation comparable to that in the liver (AA versus CO, ^*∗∗∗*^ *p* < 0.001; Figure 3(b)). Interestingly, both MTX and N-f-5HT exhibited a significant and remarkably stronger inhibition of IL-1*β* mRNA expression in comparison to that in the liver (AA-MTX versus AA, ^++^ *p* < 0.01; AA-N-f-5HT versus AA, ^+++^ *p* < 0.001; Figure 3(b)). In the spleen of N-f-5HT treated rats, the relative mRNA expression decreased even to control level.

Besides other events, MTX treatment leads to suppression of NF-*κ*B, a heterodimer consisting of two subunits p65 and p50, one of the most prominent inflammatory transcription factors activated in RA [64]. This was confirmed in our previous work, along with the finding that also N-f-5HT (15 mg/kg) suppressed the activation of NF-*κ*B (p65) in the arthritic rat liver [21, 33]. Interestingly, combination therapy (MTX + N-f-5HT) potentiated the effect of a single drug [33], suggesting different mechanisms leading to NF-*κ*B inhibition. MTX driven reduction of cytokine transcription was attributed to abrogation of I*κ*B*α* kinase activation and thereby suppression of I*κ*B*α* (NF-*κ*B inhibitor) phosphorylation and degradation, resulting in retaining the inactive NF-*κ*B form in cytoplasm [70]. However, the contribution of N-f-5HT to NF-*κ*B pathway suppression needs to be further investigated.

Studies of the proposed pathways involved in the transcription of TNF-*α*, IL-1*β*, and iNOS in RA could help evaluate the mechanism of action of these drugs [6–8, 71, 72]. The gene expression of iNOS is mostly under the control of synergistically activating NF-*κ*B (IL-1*β* and TNF-*α* stimulated) and STAT1 (IFN-*γ* stimulated) key proinflammatory signals in the liver [6]. In contrast to iNOS, TNF-*α* does not contain the STAT binding element in its promoter region. Hence, its expression is under the control of NF-*κ*B and AP-1 [7]. The inhibition of TNF-*α* and iNOS transcription observed in our study might be mostly attributed to the suppressed NF-*κ*B pathway for both MTX and N-f-5HT [33, 64, 70]. However, the contribution of AP-1 to TNF-*α* and STAT1 for iNOS cannot be excluded.

MTX-dependent suppression of NF-*κ*B was reported [33, 70, 73, 74], but in other cases MTX was not found to be effective in the attenuation of arthritic-increased mRNA expression of IL-1*β* [68, 75]. Taking into account our results, where MTX treatment did not lead to inhibition of IL-1*β* mRNA expression in the arthritic liver in contrast to the significant N-f-5HT impact, yet treatment of both MTX and N-f-5HT decreased the presumably NF-*κ*B-dependent LOX activity and iNOS and TNF-*α* transcription to a similar extent, the involvement of N-f-5HT in another pathway for transcription regulation of this cytokine in the arthritic liver should be considered. After analysis of the reported pathways involved in the regulation of IL-1*β* mRNA expression, we hypothesized that TNF-*α*-driven AP-1 transcription factor activation or JAK/STAT3 pathway activated via IL-6 or IFN-*γ* might play a role ([7, 8, 71, 72, 76, 77], Figure S1 in Supplementary Material available online at http://dx.doi.org/10.1155/2016/7509653). Papers reporting involvement of other polyphenols in anti-inflammatory regulation, for example, resveratrol, claim that these compounds exhibit their anti-inflammatory effect through suppression of NF-*κ*B and JAK/STAT signaling pathways [78, 79].

The enhanced influence of MTX and N-f-5HT on IL-1*β* transcription in the spleen in comparison to the liver may be the consequence of different predominance of inflammatory pathways in this organ, presumably with a stronger NF-*κ*B contribution. Details about the relevance of these pathways and the role of N-f-5HT in the transcription regulation of IL-1*β*, iNOS, and TNF-*α* in the liver and other organs in RA are to be further elucidated.

## 4. Conclusions

The present study contributed additional evidence about the beneficial effect and mechanism of action of N-f-5HT and of MTX on a systemic inflammatory process in the liver and its association with the pathogenesis of adjuvant arthritis. N-f-5HT treatment led to amelioration of inflammatory parameters tested (plasmatic CRP and IL-1*β* protein levels, liver LOX activity, and liver and spleen cytokine expression). However, this did not result in a significant change of HPV, although a trend of improvement of the arthritic score was observed after 28 days. Chronic inflammation is an important mediator of weight loss in the model of AA [80]. A synergistic effect of TNF-*α* and IL-1*β* was shown to influence the balance between protein degradation and protein synthesis causing among others an increase in resting energy expenditure and net efflux of amino acids from muscle to liver [81]. The significant increase of BWC in N-f-5HT treated rats, probably sign of the partial improvement of rheumatoid cachexia, might be the result of lowered mRNA expression of TNF-*α* and IL-1*β* determined in the arthritic liver. Moreover, taking into account the reported association of weight loss with the IL-1*β* production by splenic cells [80], the N-f-5HT mediated attenuation of increased IL-1*β* mRNA expression in the arthritic spleen might contribute to this complex process. The contribution of the affected expression of TNF-*α* and IL-1*β* originating from other organs cannot be excluded and is to be further elucidated.

Unexpectedly, chronic daily treatment with a high concentration of N-f-5HT (30 mg/kg) exhibited either a minor effect on the parameters examined and/or a strong variation among the animals (not shown) and that in contrast to a much lower concentration (3 mg/kg). Since N-f-5HT possesses a serotonin (5-hydroxytryptamine, 5-HT) moiety, the question if there might be some interplay between effects of these two molecules on RA pathogenesis is to be raised. Since N-f-5HT inhibited the increase of cytosolic free Ca^2+^ concentration in rat vascular smooth muscle cells induced by serotonin mediated by 5-HT_2_ receptors, it was hypothesized that at a sufficient concentration N-f-5HT may act as a competitive antagonist, which displaces serotonin from its binding site [82]. Intake of a high concentration of a 5-HT_2_ receptor antagonist may lead to a variety of effects: it may influence the receptor density, even enhance the effect of serotonin, or lead to desensitization and with time to receptor resistance (through inhibitory feedback due to binding-induced enhanced production of serotonin) [83]. Interestingly, serotonin is known not only as a neurotransmitter. Increasing but contradictory reports associate serotonin with immunoinflammatory pathways in the periphery [84]. Serotonin,* via* its 5-HT_2A_, 5-HT_2B_, and 5-HT_3_ receptors, has been implicated to have both proinflammatory and anti-inflammatory roles in a number of studies of rheumatoid arthritis [84–88]. The reported effects of 5-HT receptor antagonist on macrophage-like synovial cells encourage the interest to study the effect of N-f-5HT from this point of view [89]. To confirm this hypothesis, a precise characterization of interaction between N-f-5HT and 5-HT receptors is to be done.

On comparing the effects of the two drugs, administration of MTX (0.4 mg/kg) or N-f-5HT (3 mg/kg) was found to lead to a decrease of the main plasma marker of systemic inflammation CRP, the liver origin protein, and to inhibition of proinflammatory LOX in the liver. The impact of MTX and N-f-5HT on mRNA expression of TNF-*α*, IL-1*β*, and iNOS in the liver and on the level of CRP in plasma was mentioned at the conference [90]. MTX and N-f-5HT reduced the arthritis-increased transcription of TNF-*α* and iNOS in the liver to a comparable extent [90]. We suppose that the inhibition of TNF-*α* and iNOS transcription might be mostly attributed to the suppressed NF-*κ*B pathway for the two drugs [21, 33, 70]. As previously reported [67, 68] and also proven by our study, MTX was not able to diminish the arthritic-induced IL-1*β* mRNA transcription in the liver [90]. This handicap might be compensated by coadministration of N-f-5HT, since this drug was shown to lower the level of proinflammatory cytokine IL-1*β* in plasma in the acute phase of AA and to attenuate significantly the elevation of IL-1*β* mRNA expression in the arthritic rat liver and spleen in the chronic phase. Detailed studies are required to confirm the hypothesis that N-f-5HT might function through potentially different mechanisms of inhibition of the inflammatory pathway NF-*κ*B and not through MTX, as well as the possibility of an additional pathway influencing IL-1*β* transcription under control of N-f-5HT but not MTX. The confirmation would support N-f-5HT as a promising agent for the treatment of RA in combination therapy with MTX. The positive effect was shown in our previous study, where N-f-5HT markedly potentiated the therapeutic effect of low-dose MTX [33]. As the therapeutic dose of MTX was used in this study and the purpose of combination study is to lower the MTX dose to decrease the side effects of this drug, the effect of combination therapy was not included.

Oral daily intake of N-f-5HT could overcome the inconvenient administration and high costs of biological therapy using IL-1*β* monoclonal antibody, which was shown in clinical trials to be superior to placebo in combination with MTX in reducing signs, symptoms, and radiographic progression in patients with advanced RA [91, 92]. Future studies of N-f-5HT mechanisms of action should shed more light on the immunomodulatory function of this natural polyphenol. It is to be expected that N-f-5HT is able to positively affect the activity of other markers of inflammation and oxidative stress not only in the liver and spleen but also in other organs (lung, brain, etc.), a hypothesis to be tested by future work. However, to establish the optimal dosing in light of the effects achieved is of primary importance.

## Supplementary Material

Figure S1 Simplified overview of prominent pathways proposed to be involved in the transcription regulation of TNF*α*, iNOS and IL-1*β* in rheumatoid arthritis with the suggested effect of N-f-5HT. Binding of pro-inflammatory cytokines IL-1*β* and TNF-*α* to their respective competent receptors on target cells, IL1R1 and TNFR1, activate NF-*κ*B and MAPKs pathways. MAPKs are regulated by several upstream phosphorylation cascades. Major MAPK families involved in the response to pro-inflammatory signals are the c-JUN *N*-terminal kinases (JNKs), ERK1/2 and the p38 enzymes. Similarly, binding of IL-6 or IFN-*γ* is activating the JAK-STAT pathway. Activated transcription factors: NF-*κ*B for NF-*κ*B pathway, AP-1 for MAPKs and STAT1 or STAT3 for JAK-STAT pathway translocate from the cytoplasm into the nucleus, where they bind to the promoters of responsive genes coding for various cytokines (including TNF-*α* and IL-1*β*) and other inflammatory molecules to activate the transcription. The mRNA expression of TNF-*α* is proposed to be mostly under control of NF-*κ*B and AP-1, the expression of iNOS under control of NF-*κ*B and STAT1, and the expression of IL-1*β* under control of NF-*κ*B, AP-1 and STAT3. MTX is blocking the binding of IL-1*β* to IL1R. Both MTX and N-f-5HT have the potential to suppress NF-*κ*B activation, MTX by well characterized mechanism of inhibition of I*κ*B phosphorylation and subsequent release from the NF-*κ*B complex. Both MTX and N-f-5HT inhibit the transcription of TNF-*α* and iNOS. In addition, N-f-5HT attenuates the transcription of IL-1*β*, presumably through STAT inhibition. IL, interleukin; TNF-*α*, tumor-necrosis factor *α*; IL1R, IL-1*β* receptor; TNFR1, TNF-*α* receptor; NF-*κ*B, nuclear factor-*κ*B; MAPK, mitogen-activated protein kinase; ERK1/2, extracellular signal-regulated kinases; IFN-*γ*, interferon gamma; AP-1, activator protein-1; JAK, Janus kinase; STAT, signal transducers and activators of transcription; mRNA, messenger RNA; iNOS, inducible NO synthase; P, phosphorylation; N-f-5HT, N-feruloylserotonin; MTX, methotrexate; I*κ*B, inhibitor of NF-*κ*B.

## Figures and Tables

**Table 1:** Composition of the crystalline N-feruloylserotonin complex fraction, where the content of N-feruloyl- and N-isoferuloyl- (E = trans- and Z = cis-) serotonin isomers was determined by HPLC analysis.

Compound	Content [%]
N-(E)-Feruloylserotonin	18.3
N-(E)-Isoferuloylserotonin	67.4
N-(Z)-Feruloylserotonin	6.1
N-(Z) Isoferuloylserotonin	8.2

**Table 2:** Primer sequences.

Product	Sense primer (5′-3′)	Antisense primer (5′-3′)
IL-1*β*	CCTCTGTGACTCGTGGGATG	GGGTGTGCCGTCTTTCATCA
TNF-*α*	CTTCTGTCTACTGAACTTCG	GAACCTGGGAGTAGATAAGG
iNOS	AAAACCCCAGGTGCTATTCCC	GTGGTGAAGGGTGTCGTGAA
*β*-actin [34]	CCGCGAGTACAACCTTCTTG	GCAGCGATATCGTCATCCA

**Table 3:** Parameters of cachexia, liver weight, and severity of arthritis (hind paw volume and arthritic score) in rats with adjuvant-induced arthritis on experimental days 14 and 28 treated with N-f-5HT and MTX.

Cachexia	CO	AA	AA-MTX	AA-N-f-5HT
*Parameter*				
*Severity of arthritis*				
Hind paw volume				
Day 14	1.67 ± 0.01	1.82 ± 0.04	1.69 ± 0.03	1.87 ± 0.06
Day 21	1.78 ± 0.02	2.07 ± 0.08^*∗∗*^	1.72 ± 0.04^+++^	2.07 ± 0.05
Day 28	1.81 ± 0.02	1.98 ± 0.07^*∗*^	1.72 ± 0.03^+++^	1.95 ± 0.04

Arthritic score				
Day 14	10 ± 0	15.1 ± 0.79^*∗∗*^	12.4 ± 0.72	16.7 ± 1.36
Day 21	11.25 ± 0.32	19.71 ± 1.11^*∗∗∗*^	14.4 ± 1.68^+^	19.6 ± 1.37
Day 28	11.37 ± 0.26	22.29 ± 0.71^*∗∗∗*^	16.3 ± 1.11^++^	19.5 ± 1.23

*Cachexia*				
Body weight change (g)				
Day 14	59.37 ± 1.80	33.25 ± 4.80^*∗∗∗*^	42.82 ± 3.74	39.92 ± 5.33
Day 21	75.28 ± 2.68	14.14 ± 4.97^*∗∗∗*^	31.15 ± 6.17	29.6 ± 4.52
Day 28	102.69 ± 4.32	28.1 ± 4.61^*∗∗∗*^	38.9 ± 8.03	55.32 ± 4.99^+^

Liver weight (g)				
Day 28	7.94 ± 0.17	7.99 ± 0.21	7.05 ± 0.23^+^	8.32 ± 0.23

CO, control group; AA, adjuvant arthritis group; AA-N-f-5HT, adjuvant arthritis group given N-feruloylserotonin; AA-MTX, adjuvant arthritis group given methotrexate. The data represent the mean ± SEM; *n* = 9-10. The symbols *∗* and + show significant difference: ^*∗*^ *p* < 0.05 versus CO; ^*∗∗*^ *p* < 0.01 versus CO; ^*∗∗∗*^ *p* < 0.001 versus CO; ^+^ *p* < 0.05 versus AA; ^++^ *p* < 0.01 versus AA; ^+++^ *p* < 0.001 versus AA.
