# Supplementary material for: Effect of N-Feruloylserotonin and Methotrexate on Severity of Experimental Arthritis and on Messenger RNA Expression of Key Proinflammatory Markers in Liver
Source: J Immunol Res. 2016 Jul 31;2016:7509653. doi: 10.1155/2016/7509653 (PMC4983360; doi:10.1155/2016/7509653)

Supplementary data:

Figure S1 Simplified overview of prominent pathways proposed to be involved in the transcription regulation of TNFα, iNOS and IL-1β in rheumatoid arthritis with the suggested effect of N-f-5HT. Binding of pro-inflammatory cytokines IL-1β and TNF-α to their respective competent receptors on target cells, IL1R1 and TNFR1, activate NF-κB and MAPKs pathways. MAPKs are regulated by several upstream phosphorylation cascades. Major MAPK families involved in the response to pro-inflammatory signals are the c-JUN *N*-terminal kinases (JNKs), ERK1/2 and the p38 enzymes. Similarly, binding of IL-6 or IFN-γ is activating the JAK-STAT pathway. Activated transcription factors: NF-κB for NF-κB pathway, AP-1 for MAPKs and STAT1 or STAT3 for JAK-STAT pathway translocate from the cytoplasm into the nucleus, where they bind to the promoters of responsive genes coding for various cytokines (including TNF-α and IL-1β) and other inflammatory molecules to activate the transcription. The mRNA expression of TNF-α is proposed to be mostly under control of NF-κB and AP-1, the expression of iNOS under control of NF-κB and STAT1, and the expression of IL-1β under control of NF-κB, AP-1 and STAT3. MTX is blocking the binding of IL-1β to IL1R. Both MTX and N-f-5HT have the potential to suppress NF-κB activation, MTX by well characterized mechanism of inhibition of IκB phosphorylation and subsequent release from the NF-κB complex. Both MTX and N-f-5HT inhibit the transcription of TNF-α and iNOS. In addition, N-f-5HT attenuates the transcription of IL-1β, presumably through STAT inhibition.

IL, interleukin; TNF-α, tumor-necrosis factor α; IL1R, IL-1β receptor; TNFR1, TNF-α receptor; NF-κB, nuclear factor- κB; MAPK, mitogen-activated protein kinase; ERK1/2, extracellular signal-regulated kinases; IFN-γ, interferon gamma; AP-1, activator protein-1; JAK, Janus kinase; STAT, signal transducers and activators of transcription; mRNA, messenger RNA; iNOS, inducible NO synthase; P, phosphorylation; N-f-5HT, N-feruloylserotonin; MTX, methotrexate; IκB, inhibitor of NF-κB.

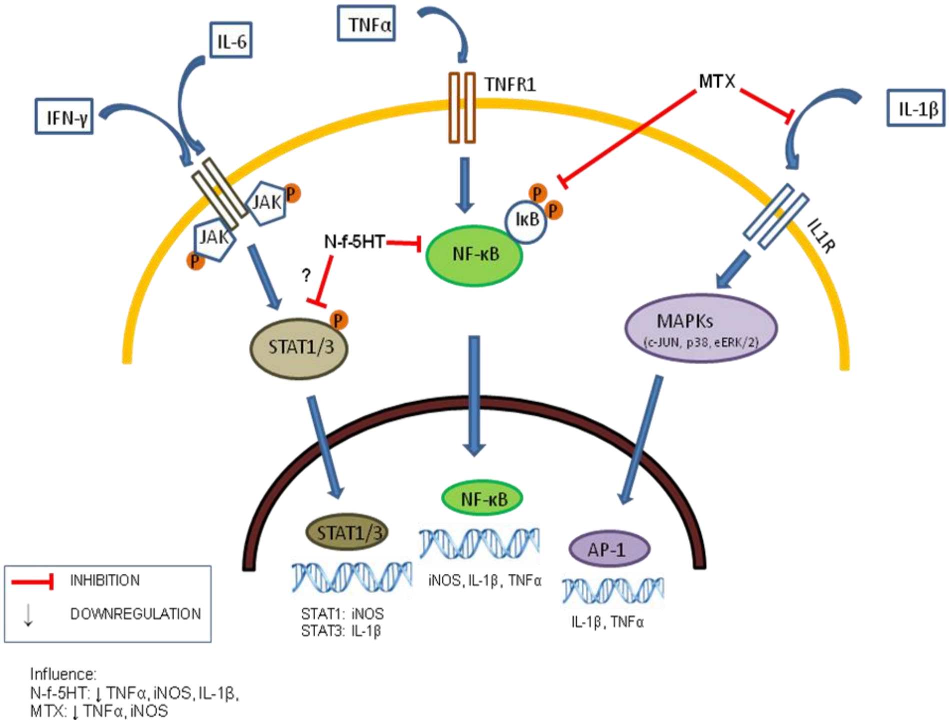

Supplement: Supplementary file 1 — Figure S1 Simplified overview of prominent pathways proposed to be involved in the transcription regulation of TNFα, iNOS and IL-1β in rheumatoid arthritis with the suggested effect of N-f-5HT. Binding of pro-inflammatory cytokines IL-1β and TNF-α to their respective competent receptors on target cells, IL1R1 and TNFR1, activate NF-κB and MAPKs pathways. MAPKs are regulated by several upstream phosphorylation cascades. Major MAPK families involved in the response to pro-inflammatory signals are the c-JUN N-terminal kinases (JNKs), ERK1/2 and the p38 enzymes. Similarly, binding of IL-6 or IFN-γ is activating the JAK-STAT pathway. Activated transcription factors: NF-κB for NF-κB pathway, AP-1 for MAPKs and STAT1 or STAT3 for JAK-STAT pathway translocate from the cytoplasm into the nucleus, where they bind to the promoters of responsive genes coding for various cytokines (including TNF-α and IL-1β) and other inflammatory molecules to activate the transcription. The mRNA expression of TNF-α is proposed to be mostly under control of NF-κB and AP-1, the expression of iNOS under control of NF-κB and STAT1, and the expression of IL-1β under control of NF-κB, AP-1 and STAT3. MTX is blocking the binding of IL-1β to IL1R. Both MTX and N-f-5HT have the potential to suppress NF-κB activation, MTX by well characterized mechanism of inhibition of IκB phosphorylation and subsequent release from the NF-κB complex. Both MTX and N-f-5HT inhibit the transcription of TNF-α and iNOS. In addition, N-f-5HT attenuates the transcription of IL-1β, presumably through STAT inhibition. IL, interleukin; TNF-α, tumor-necrosis factor α; IL1R, IL-1β receptor; TNFR1, TNF-α receptor; NF-κB, nuclear factor-κB; MAPK, mitogen-activated protein kinase; ERK1/2, extracellular signal-regulated kinases; IFN-γ, interferon gamma; AP-1, activator protein-1; JAK, Janus kinase; STAT, signal transducers and activators of transcription; mRNA, messenger RNA; iNOS, inducible NO synthase; P, phosphorylation; N-f-5HT, N [file 7509653.f1.pdf]
